# The Management of Children’s Food Allergy in Childcare Centres, Preschools, and Schools: A Scoping Review

**DOI:** 10.3390/nu17172722

**Published:** 2025-08-22

**Authors:** Prathyusha Sanagavarapu, Sainiana Rika, Constance H. Katelaris, Maria Said, Lily Collison, Ann Dadich

**Affiliations:** 1 School of Education, Translational Health Research Institute (THRI), Transforming Early Childhood Health and Education (TeEACH), Western Sydney University, Penrith, NSW 2751, Australia; 2 St Luke’s Catholic College, Sydney, NSW 2765, Australia; sainianahicks@gmail.com; 3 Immunology & Allergy Unit, Department of Medicine, Campbelltown Hospital, Sydney, NSW 2560, Australia; connie.katelaris@health.nsw.gov.au; 4 Allergy & Anaphylaxis Australia, Sydney, NSW 2154, Australia; msaid@allergyfacts.org.au; 5 Medical and Health Sciences Librarian, Western Sydney University, Penrith, NSW 2751, Australia; l.collison@westernsydney.edu.au; 6 School of Business, Western Sydney University, Penrith, NSW 2751, Australia; a.dadich@westernsydney.edu.au

**Keywords:** children’s food allergy, schools, childcare centres, scoping review, food allergy safety, psychological support

## Abstract

Background: There are very few reviews on how children’s food allergy is managed across various educational settings, and none have considered psychological support in addition to child safety. This scoping review aimed to understand interventions to manage food allergy, addressing children’s safety and psychological support in childcare centres, preschools, and schools. Methods: Following the JBI methodology for scoping reviews and applying the PCC (population, concept, and context) mnemonic, a search was conducted via Medline (Ovid), Embase (Ovid), CINAHL (EBSCOhost), ERIC (ProQuest), PsychInfo (EBSCOhost), Scopus, and ProQuest Dissertations and Theses (ProQuest). Furthermore, two supplementary searches were conducted: first, backward citation tracking of all publications included in this review; and second, a search of seven peak allergy organisation websites, including Allergy & Anaphylaxis Australia and the World Allergy Organization. Findings: Eighteen publications were included from 6812 records retrieved from the databases. Most publications were from the United States of America (61%), representing food allergy management mainly in schools (39%), followed by preschools (22%), childcare centres (17%), and mixed settings (11%). All the interventions focused on child physical safety, largely neglecting psychosocial support for children or their families, and only four publications reported the use of control groups to test intervention benefits (22%). Furthermore, safety-focused interventions were centred on building educator or staff knowledge of food allergy and their skills, confidence, and self-efficacy to manage it (72%); these were found to be highly effective. Most interventions were aimed at adults, and none considered children. Interpretation: The findings suggest a need for more research on food allergy management involving child-focused, developmentally appropriate approaches, especially in childcare and preschool settings. There is also a need for research on psychological support, particularly that which involves control groups and encompasses different nations.

## 1. Introduction

A growing number of infants and children worldwide are experiencing food allergy [1,2]—that is, a specific, reproducible, immune response to a particular food allergen. For instance, an Australian study found that over ten per cent of one-year-old infants [3] and six per cent of six-year-olds were affected by food allergy [4].

Children’s food allergy can have considerable personal, social, organisational, and economic consequences. At a personal level, children can develop severe allergic reactions [5,6], and although rare, fatal anaphylaxis [7]; feel isolated [8]; and experience bullying [9,10] and anxiety [11]. At a social level, food allergy can exacerbate families’ burden of care [12]. Specifically, food allergy is associated with parental distress and functional impairment [13]. Additionally, the psychosocial burden and quality of life issues for affected children and families are well-documented [14]. For organisations, food allergy can strain limited resources, including emergency services [15], treatments, therapies, and staff time, which in turn has economic implications. For instance, given the need for primary care, specialist care, tests, and prescriptions, food allergy among children aged one to four years in Australia was reported to cost Australia’s universal health insurance scheme AUD 889.70 per child; furthermore, projections estimated a cost of AUD 26.1 million in 2020 [16].

When children with food allergy transition from home to childcare centres, preschools, and schools, the school community—including centre directors, educators, principals, school nurses, administrative staff, and parents—becomes responsible for ensuring these children remain safe in these settings [17,18]. In the United States of America (USA), food allergy in educational settings is managed by dealing with each child’s allergy; preparing staff members to manage food allergy emergencies; educating children and family members about food allergy; and maintaining safe environments [19]. In England, it involves the receipt of information from food suppliers about changes to food products; checking food labels for potential allergens; and ensuring caterers list all allergens and ingredients in their dishes and menu charts [20]. In Australia, food allergy management in childcare and school settings can involve food allergy management plans; providing information on food allergens; staff training; minimising children’s exposure to food allergens; providing inclusive curricular and extracurricular activities for children; as well as developing a management plan for each child [21].

Globally, there are many policies and guidelines to manage food allergy in educational settings. These include Australia’s best practice guidelines to prevent severe allergic reactions in schools and children’s education and care settings [22]; the European Academy of Allergy and Clinical Immunology (EAACI) guidelines; the Food Allergy Management in Schools [23]; and practice guidelines to prevent and manage food allergy in childcare centres and schools [24]. These suggest there are considerable differences in how children’s food allergy is managed in schools among countries [25] and among states within the same country [24]. Given these disparities, Deschildre and colleagues [26] suggested a universally agreed approach to manage children’s food allergy in schools. They emphasised staff training, allergy prevention, preparing and managing allergic reactions, involving children, and providing food allergy education for all children in schools.

There are very few reviews on how children’s food allergy is managed across various educational settings and the associated effects [24,27,28]. This is partly because food allergy management is shaped by myriad factors, including educator or staff member knowledge, skills, and confidence, as well as the policies and practices of an educational setting [29,30,31,32]. Also, no reviews have considered psychological support in addition to child safety. Therefore, this article presents the evidence on food allergy management in childcare centres, preschools, and schools. This was achieved by conducting a scoping review to map what is known and unknown [33] about tested approaches that staff members use in these settings to promote the safety and psychological support of children with food allergy. A scoping review was appropriate for three key reasons. First, the participants, concept, and context are ill-defined, whereby there are no universal understandings of what constitutes children who attend childcare centres and preschools, the management of their food allergy, or childcare centres and preschools. As such, although relevant academic databases were systematically searched, a scoping review was required to map the literature [34]. Second, a scoping review typically precedes a systematic review to establish the strategies that are effective [34]. Third, and relatedly, governments have a demonstrated interest in ensuring the safety of children with food allergy in educational settings, and information from a scoping review can assist with this [19,20,21].

## 2. Materials and Methods

### 2.1. Search Strategy

This scoping review was conducted in accordance with the JBI methodology for scoping reviews, including protocol registration, searches, and data extraction, synthesis, and reporting [35]. However, educators, families, school nurses, and other school staff were not involved in the review [36] due to resource constraints.

A systematic search of Medline (Ovid), Embase (Ovid), CINAHL (EBSCOhost), ERIC (ProQuest), PsychInfo (EBSCOhost), Scopus, and ProQuest Dissertations and Theses (ProQuest) was conducted. Initially, search terms were identified by applying the PCC (population, concept, and context) mnemonic [35]. Publications were included if they included the population of interest—namely, children, aged 0 to 12 years; the concept of interest—namely, food allergy management; and the context of interest—namely, childcare centres, preschools, and schools. A list of terms for the population, concept, and context was developed, and an initial search was conducted using the Medline (Ovid) database. The resulting records were scanned for index and additional terms. The Medline thesaurus was also searched to locate index terms.

A second preliminary search was conducted using the Medline title, abstract, and index term fields. Boolean operators, truncations, and database-specific proximity operators were applied to operationalise the search to increase the sensitivity of the results. This preliminary search strategy was translated into and deployed in the Embase database. The Medline search strategy was then amended to include additional terms identified from the Embase search. One author—a librarian—then deployed this enhanced search strategy across all the selected databases. The search strategy was adjusted (using free text and index terms) to match the requirements of each database. Publications were included if they were published in English (given that the accuracy of translation platforms, like Google Translate, could not be verified), irrespective of publication date (for inclusiveness).

Two additional supplementary searches were conducted. The first involved backward citation tracking of all publications included in this review, where one author (PS) scanned the reference lists to locate additional, relevant publications. Second, subject matter experts within the team (CK, MS, PS) identified eight relevant peak organisations [37,38,39,40,41,42,43,44]. Two authors (PS, SR) searched the websites and webpages of these organisations for additional, relevant publications. These two supplementary searches did not identify additional publications for this review. The search results from the databases were imported into EndNote 20 [45] on 23 September 2023 (see Appendix A for a copy of the Medline search strategy).

The review protocol was registered on the Open Access Framework on 5 February 2023 (Registration: https://doi.org/10.17605/OSF.IO/C9E6Z). Following this, the inclusion criterion of ‘an approach or strategy to manage children’s food allergy addressing safety and psychological support’ was changed to ‘a tested approach or strategy to manage children’s food allergy addressing safety and psychological support.’ This served to include only interventions that had been tested to understand their efficacy.

### 2.2. Data Selection, Management, and Analysis

Once duplicates were removed using EndNote, the remaining records were imported into the Covidence software [46] to manage the review. Two authors (PS, SR) screened the titles and abstracts of all publication records to determine whether they met the inclusion criteria. Of those that did, these authors sourced and reviewed the full text. Discrepancies were resolved through discussion with two authors (CK, AD) with content expertise. Two authors (PS, SR) extracted data from the relevant publications and reported the process according to the Preferred Reporting Items for Systematic Reviews and Meta-Analyses extension for scoping reviews (PRISMA-ScR) checklist [47]; discrepancies were resolved by a third author (AD). Extracted data were summarised (see Table 1). When the population, concept, or context was unclear in a potentially relevant publication, the lead author of the publication was contacted to request this information and provided with two weeks to respond—failing this, the publication was excluded. When a publication presented information on the population, concept, or context that was aggregated with information that was beyond the scope of this review, relevant data were disaggregated when feasible. If this was not feasible, the publication was excluded. The publications that warranted inclusion in this review were not appraised for quality, as per the scoping review guidelines [48]. Given there is no universally agreed approach to identify the unique studies reported across the identified publications [49], this scoping review reports on the content of the publications, rather than the studies reported across them.

The data extracted from the publications were reviewed and synthesised (PS). This involved categorising the data by author(s), country, year, setting, sample, methods, control group (see Table 1). The tested approaches or strategies to manage children’s food allergy are categorised into those addressing safety or psychosocial support. Safety approaches or strategies were those that served to prevent allergy or an allergic reaction (e.g., handwashing, banning foods, avoiding food sharing); build educator or staff member knowledge, skills, and confidence to manage allergy and anaphylaxis (e.g., identify allergy-causing foods, recognise symptoms, manage and treat allergic reactions, administer medications); and improve the management of anaphylaxis and allergic reactions (e.g., revising or developing policies, guidelines, or practices). Psychosocial support approaches or strategies were those that served to build knowledge or understanding about children’s or families’ psychosocial needs, and support their well-being (e.g., addressing or preventing potential discrimination). The data extraction strategy was piloted and cross-checked with the team members (SR, CK, AD, MS) (see Table 2). Moreover, the approaches used to manage food allergy were categorised by the type of setting. See Table 3. 

## 3. Results

**Figure 1:**
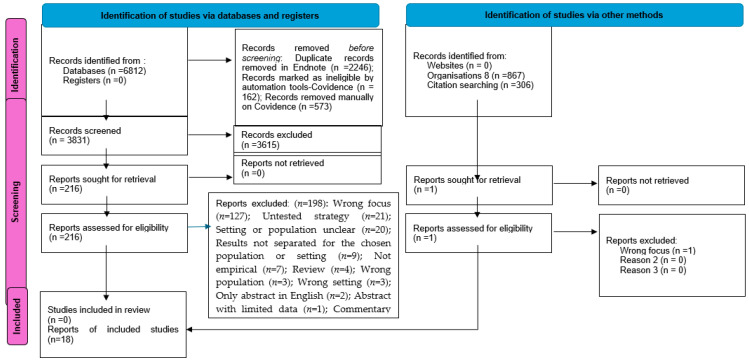
PRISMA 2020 flow diagram for new systematic reviews that included searches of databases, registers, and other sources.

Following the identification of 6812 publications, 18 were deemed relevant and included in this review (see Figure 1).

As indicated in Table 1, eleven of the 18 publications reported on research conducted in the USA (61.0%); three in Italy (17.0%); and one each in Canada, Japan, Spain, and the United Kingdom (22%). They were published between 2005 and 2021, inclusive, in journals (99.0%), except for one, which was an unpublished doctoral thesis (1.0%). The settings represented in the publications included 11 schools (61.5%), 4 preschool or early childhood settings (22.0%), and 3 childcare centres (16.5%). Eight publications involved a single participant group, either parent–child dyads [66], school nurses [52], childcare workers [57,58], preschool teachers [54,56], or school teachers [62,65]. The remaining publications involved a mix of participants, including school principals [59], head teachers or nominees [61], centre directors [51,53], dietitians [50], school administrative staff members and caretakers [60], home visitors [55], nurses [64], canteen staff [63], camp counsellors, bus drivers, coaches, food service personnel, as well as staff members from museum and government agencies [67]. All publications reported the collection and analysis of quantitative data, such as questionnaires, except for two publications, which reported on mixed methods, including questionnaires with an inspection of school lunches [50] or focus groups [62]. Only four publications reported the involvement of a control group to test the benefits of an intervention to manage children’s food allergy and address safety and/or psychosocial support (22.0%) [50,52,65,66] (see Table 1).

Further, as indicated in Table 2, all the interventions presented in the publications focused on child physical safety, largely neglecting psychosocial support for children or their families (see Table 2). The safety interventions included efforts to prevent children’s exposure to allergens; ban foods (11.0%); educate staff members on food allergy management (72.0%); review food allergy policy (0%); and combined approaches (17.0%). The durations of the reported interventions were less than an hour [53,54,55,57,62,65,67]; two hours [59,60,61,63]; one to three months [66]; six to twelve months [50]; or three to five years [52]. However, the duration was unspecified in some publications [51,56,58,64].

### 3.1. Child Safety

#### 3.1.1. Preventing Food Allergy and Reactions

Two publications (11.1%) reported on banning foods in schools, reflecting relevant policies or guidelines. Adopting a mixed-methods and control group design, Banerjee and colleagues [50] inspected children’s lunches in kindergarten to grade three classes in schools with and without peanut-free guidelines. They also surveyed parents, teachers, and principals on policy implementation. A dietitian randomly inspected 854 school lunches over approximately 94.5 days to ascertain parental awareness of and adherence to peanut-free guidelines. The findings suggested that the lunches inspected in classrooms with peanut-free guidelines contained substantially fewer peanuts (0.6%, 95% CI 0.2% to 1.4%) than in those without peanut-free guidelines (9.9%, 95% CI 8.0% to 12.2%)—representing a 9.4% difference (95% CI 7.3% to 11.4%). Bartnikas and colleagues [52] compared the rates of epinephrine (also known as adrenaline) administration in 484 Massachusetts public schools with and without peanut-restrictive policies over five years. They found that policies restricting peanuts from home, served in schools, or brought into classrooms did not affect the rates of epinephrine administration. Conversely, schools with peanut-free lunch tables had lower rates of epinephrine administration compared to those without (incidence rates of 0.2 and 0.6 per 10,000 students, respectively; *p* = 0.009).

#### 3.1.2. Building Knowledge, Skills, and Confidence

Most publications (72%) reported on building educators’ or staff members’ knowledge, skills, and confidence in managing and treating food allergy. This was the most preferred intervention across childcare centres (17%), preschools (22%), schools (28%), and combined settings (11%). See Table 3. Food allergy education was similar across all settings. For example, in childcare centres, educators were provided with a food allergy curriculum [57] or allergy seminars [51,58]. Similarly, in preschools, staff members were provided with in-service training on: food allergy, including food labelling [53]; the administration of the epinephrine autoinjector, allergies, anaphylaxis, and emergencies [54,56]; and anaphylaxis recognition and management [55]. In schools, food allergy education included knowledge and skills in allergy recognition, avoidance, and management [62,63,64,65,67], as well as a child-targeted educational booklet on food allergy [66].

Subject matter experts—including paediatric allergists, paediatricians, pharmacists, and lawyers—typically developed the training materials used to build staff knowledge, skills, and confidence as reported in all publications. While these materials were largely developed for adults, one publication reported the use of the training materials by children and their family members [66].

While most educational efforts involved a single seminar or workshop, Ravarotto and colleagues [62] adopted a multipronged approach. This included three focus groups with 25 teachers to examine teachers’ perceptions, knowledge, and information needs. This was followed by five workshops to provide teachers with targeted scientific information, a forty-minute presentation for teachers, and its evaluation. The impact of the interventions to build knowledge, skills, and confidence was largely determined by collecting pre- and post-educational session quantitative data, often via a questionnaire. Most interventions focused on changes in participants’ skills, attitudes, and knowledge on food allergy, symptom recognition, and treatment protocols [51,53,54,55,57,58,62,63,64,65,66,67].

All the publications reported the benefits associated with interventions to build knowledge, skills, and confidence. For instance, Crow [53] noted that a forty-minute in-service training session enhanced preschool educators’ and staff members’ awareness about food allergy, anaphylaxis, emergency action plans, the epinephrine autoinjector, and related guidelines. Specifically, the pre- and post-educational session test scores significantly improved, whereby 29% of the participants scored less than 50% on their food allergy knowledge before the training, and 71% scored more than 50% after the training. Similarly, Ravarotto and colleagues [62] demonstrated a 72% increase in participants’ correct responses to questions on food allergy, irrespective of their age (χ^2^ = 6.1888, *p* = 0.0402) or previous training on food allergy (χ^2^ = 0.143, *p* = 0.931).

Two publications reported on the use of control groups to evaluate interventions to build knowledge, skills, and confidence. For example, Shroba and McElroy [66] tested the efficacy of a parent and child educational booklet on food allergy. This involved randomly providing the booklet to 29 children, aged 5 to 11 years, and their carers, and assessing their knowledge of food allergy using a questionnaire. The findings suggested that child-based education was equally valuable in promoting food allergy knowledge (*t* = 1.782, *p* = 0.089) and safety (χ^2^ = 0.524, *p* = 0.47) when compared to carer-focused education. Additionally, Shah and colleagues [65] showed improvements in teacher knowledge of causative foods, symptoms, and the treatment of reactions in elementary schools.

While all publications demonstrated the benefits associated with educational interventions, three publications noted that benefits could decrease over time For example, Bansal and colleagues [51] showed a significant decrease in childcare staff member knowledge of when to administer an adrenaline autoinjector at six months (48%, *p* = 0.02) and one year (38%, *p* = 0.002). Dumeier and colleagues [54] also showed a decrease in educator preparedness to manage anaphylaxis from 88% to 79% over 4 to 12 weeks post-training (each *p* < 0.001). Similarly, Patel and colleagues [58] reported that, after training, only 48% and 31% of childcare staff knew how to correctly administer an adrenaline auto injector at six months (*p* = 0.02) and one year (*p* = 0.002), respectively.

#### 3.1.3. Mixed Approaches

##### Educational Intervention Combined with Psychological Support

Two publications reported on psychological support (11%), which was embedded into school educational interventions. Psychological support involved building awareness about children’s concerns and anxiety, potential exclusion from school activities, and bullying experiences. Pollini and colleagues [59] reported on the benefits of a food allergy educational session for primary school teachers—it improved their self-efficacy to manage food allergy and their knowledge of allergy. They reported that their intervention also helped to increase primary school educators’ awareness of children’s psychological concerns relating to food allergy (*f* = 13.450, *df* = 2, *p* < 0.001). According to these authors, the educators or staff members who participated in their study appreciated the importance of providing psychological support to children.

Similarly, Polloni and colleagues [11] showed improvements in school personnel’s self-efficacy relating to food allergy management—this was especially the case among those with low self-efficacy before the training. However, this intervention did not help to ensure children’s full participation in all schools’ curricular or non-curricular activities. Yet the reported differences between the pre- and post-training scores of 1.00 in the third quartile and zero in the first quartile were statistically insignificant.

##### Educational Intervention Combined with Policy Review 

One publication addressed safety and policy review topics in its educational intervention (6%). Raptis and colleagues [61] provided a ninety-minute educational session for directors of childcare centres that combined knowledge and skills on food allergy management with the development of allergy action plans, allergy management, health care plans, and regulations. It served to build head teachers’ knowledge and confidence from 39% to 83% (*p* = 0.016). It also helped to improve the centres’ food allergy policies—directors were able to review their current policies, ensuring these promoted awareness training for children and prevented children’s exposure to allergic foods. They reported a 100% adoption of a ‘no food sharing’ policy, post-training, from a 61% adoption of this policy before training (*p* = 0.03). The educators realised the importance of providing children with teaching material and practical skills to self-manage their allergies.

## 4. Discussion

This scoping review synthesised the evidence on interventions to manage food allergy, addressing both safety and psychological support in childcare centres, preschools, and schools. The primary rationale for this review was the dearth of evidence on how food allergy is managed in these settings, despite the increasing prevalence of food allergy in young infants and children [1,2,3,4].

This review suggests that research on food allergy management in childcare, preschool, school, and mixed settings, mainly from the USA, has largely focused on children’s safety, while neglecting children’s and parents’ psychosocial needs. This is despite the importance of these psychosocial needs [68]. This finding reflects research that reported greater emphasis on the medical management of food allergy [69]. Only two publications reported on educational interventions to bolster psychological support. Of these, one reported on an educational intervention that did not improve educator attitudes towards children’s inclusion in school activities [59]. The other reported on an intervention that promoted educator awareness of children’s emotional concerns regarding allergy [60]. The reasons for these variations in efficacy are unclear.

Overall, safety-focused interventions across all settings centred on building educator or staff member knowledge of food allergy and their skills, confidence, and self-efficacy to manage it, and these interventions were effective. While most educational interventions aimed to build educators’ or school personnel’s knowledge and skills in food allergy management, two publications emphasised the need to build their self-efficacy [59,60]—this is because they might be apprehensive about treating anaphylaxis without healthcare training. This finding reflects research that found educational interventions can increase participant knowledge, attitudes, beliefs, and confidence to manage food allergy and anaphylaxis [27]. It also aligns with gaps in the management of food allergy in educational settings, including staff failing to identify children with food allergy; prevent children’s accidental exposure to allergens; and manage their anaphylaxis reactions [70,71].

Furthermore, the study’s findings revealed that food allergy training provided to educators by medical, health, and legal professionals was mainly focused on child safety. There is a need to integrate psychological well-being into educators’ paediatric allergy training to promote and monitor children’s well-being in educational settings, as acknowledged in the literature [14]. Also, to better monitor and support children’s holistic well-being, it is equally important to have a multi-disciplinary support team in schools globally, including school nurses, psychologists, and dietitians alongside the paediatricians and allergists.

While childcare and preschool settings relied on educational interventions to promote children’s safety, schools focused on a range of measures to promote children’s safety. The exclusive emphasis on educational interventions in childcare and preschool settings might be partly because educators in these settings are sometimes deemed to have limited knowledge of food allergy and its management [59]. They can also find it difficult to manage food allergy without the support of full-time, on-site school nurses [72]. Schools demonstrated efforts to promote children’s safety by banning foods, thereby adhering to relevant policies or guidelines. These findings indicated that managing food allergy can vary by setting type. Childcare centres, preschools, and schools differ in their student body, school community, and policies. Contextual differences can present varied challenges and strategies to manage food allergy. While this review did not explicate the contextual factors, they appear to shape food allergy management [24,73].

School policies and guidelines were not particularly effective [50,52], reflecting previous research [24]. However, these publications noted that education, supervision, and banning foods in designated areas can be effective. The two publications that reported on food bans in schools were published in the early 2000s—given the impracticality of food bans, there has since been a shift in the advice and adoption of food ban policies in childcare centres, preschools, and schools [24]. This review also did not identify publications that reported on, perhaps more practicable, preventative strategies—for instance, handwashing, which is valuable for the child with food allergy (before eating) and for children without food allergy (once they have eaten). This signals a future research area.

The inclusion of educators [54,65] and other stakeholders [67] in the educational interventions reflects the growing recognition that food allergy management is everyone’s business [17,74]. Yet all publications focused on adults and none considered children, except one that involved parent–child dyads [66]. Furthermore, the publications did not establish the appropriateness of the interventions for children of different ages, grades, or developmental needs. Therefore, there is a need for research that serves to involve children in the design and testing of interventions, and to determine how children’s developmental needs shape food allergy management.

### 4.1. Limitations

Despite the value of the findings from this review, three methodological limitations warrant mention. First, given the various terms used to refer to the population, concept, and contexts of interest, the search strategies might not have identified all relevant publications. Second, given the focus on English publications, the review might have omitted relevant publications in other languages. Third, the study’s focus on tested interventions limited the opportunities to identify untested, yet potentially effective interventions and to explore qualitative insights of food allergy management.

### 4.2. Implications for Educational Practice and Future Research

Despite the aforementioned limitations, the findings from this review have important implications for educators and scholars. For educators, the review demonstrates the feasibility of educating staff members in food allergy management to build their knowledge and skills; the importance of empowering all stakeholders to manage food allergy; and the potential value of multiple strategies to manage food allergy, corresponding with global recommendations [24,26].

For scholars, the findings from this review suggest a need for more research addressing the three main gaps identified. First, there is a need to diversify the nations in which research is conducted. For instance, to strengthen the global relevance and educational impact of this review, future research should incorporate a comprehensive international survey supported by a rigorously designed questionnaire. This would enable a comparative analysis of the situation and significance of the problem across diverse national contexts, moving beyond the current USA-centric examples. By capturing data from multiple countries, the study could illuminate how cultural, institutional, and policy differences shape the issue, thereby enriching the analysis and enhancing the validity of proposed solutions. Such an approach would not only broaden the empirical base but also facilitate the development of context-sensitive strategies that are both scalable and adaptable. Ultimately, this would contribute to a more nuanced understanding of the problem and support the formulation of educational interventions that are globally informed and locally responsive. Second, research is needed that is longitudinal and involves mixed methods to capture different forms and sources of data. Third, there is a need to involve different stakeholders in research [36], particularly children, given the demonstrated value of child-focused, developmentally appropriate educational interventions [66].

## 5. Conclusions

This is one of the first scoping reviews to synthesise interventions for manage food allergy, addressing children’s safety and psychological support in childcare centres, preschools, and schools. All the interventions in the selected publications focused on child physical safety, while largely neglecting psychosocial support for children or their families. Furthermore, the interventions varied across early childhood and school settings. While a multipronged approach was used in a school setting, childcare and preschools focused on child safety. Moreover, most research was from the USA, and only a few publications assessed the efficacy of the interventions using control groups. Future research should thus include an international questionnaire to contextualise the findings Importantly, most interventions were aimed at adults, and none considered children. These findings revealed a paucity of tested interventions to manage food allergy among children. Also, they indicated a need for more research to empower and involve different stakeholders—notably, children—in the management of their food allergy in educational settings in developmentally appropriate ways.

## Figures and Tables

**Table 1:** Publication details (Total number of publications = 18).

Publication	Country	Setting	Sample	Method/s	Control Group
[50]	Canada	School	Dietitians, parents, principals, and teachers from 97 classes with and 94 classes without peanut-free guidelines	Mixed: Inspection of school lunches and questionnaire	Yes
[51]	USA	Childcare	Teachers and directors from 44 childcare centres	Quantitative: Pre- and post-educational session questionnaire	No
[52]	USA	School	397 nurses from schools with and without a peanut policy	Quantitative: Questionnaire of schools with and without peanut-free policies	Yes
[53]	USA	Preschool	24 teachers and 2 directors	Quantitative: Pre- and post-educational session questionnaire	No
[54]	USA	Preschool	75 teachers	Quantitative: Pre- and post-educational session questionnaire	No
[55]	USA	Preschool	181 participants (directors, teachers, assistants, teaching aides, home visitors)	Quantitative: Pre- and post-educational session questionnaire	No
[56]	USA	Early childhood setting	127 early childhood professionals (roles unspecified)	Quantitative: Pre- and post-educational session questionnaire	No
[57]	USA	Childcare centre	73 childcare workers	Quantitative: Pre- and post-educational session questionnaire	No
[58]	USA	Childcare centre	39 centre directors	Quantitative: Pre- and post-educational session questionnaire	No
[59]	Italy	School	1184 teachers and principals	Quantitative: Pre- and post-educational session questionnaire	No
[60]	Italy	School	592 teachers and caretakers	Quantitative: Pre- and post-educational session questionnaire	No
[61]	United Kingdom	School	191 school personnel from 18 schools (head teachers, nominees)	Quantitative: Pre- and post-educational session questionnaire	No
[62]	Italy	School	158 teachers	Quantitative: Pre- and post-educational session questionnaire	No
[63]	Spain	School	53 teachers and canteen staff	Quantitative: Pre- and post-educational session questionnaire	No
[64]	Japan	School and childcare	110 school nurses, 78 schoolteachers (elementary school, junior high school), and 120 childcare workers	Quantitative: Pre- and post-educational session questionnaire	No
[65]	USA	School	195 teachers	Quantitative: Pre- and post-educational session questionnaire	Yes
[66]	USA	School	29 children and their families	Quantitative: Pre- and post-educational session (with a booklet) questionnaire	Yes
[67]	USA	School and community	4818 participants (preschool and childcare providers, teachers in Montessori, elementary, middle, and high schools, school aides, bus drivers, nurses, camp counsellors, government agency personnel)	Mixed: Pre- and post-educational session questionnaire and telephone interview	No

**Table 2:** Interventions to manage food allergy (Total number of publications= 18).

Domains	Main Approach	Number and Percentages	Interventions	Setting	Publication
Child safety	Preventing allergy and/or allergic reactions	2 (11.0%)	Banning foods: Adherence to peanut-free guidelines and peanut-free policy	School (2)	[50,52]
	Building educator/staff knowledge, skills, and confidence or self-efficacy	13 (72%)	Educational in-service workshops, seminars, and theoretical and practical training in the use of an adrenaline autoinjector	Preschool and early childhood (4), childcare centres (3), schools (5), and combined settings (2)	[51,53,54,55,56,57,58,62,63,64,65,66,67]
	Policy and practice improvements	0 (0.0%)			
Psychological support	Address children’s or families’ quality of life issues	0 (0.0%)			
Safety and psychological support combined	Building educator/staff knowledge and policy/practice review	1(6%)	Educational in-service training and policy/practice review	School (1)	[61]
	Building educator/staff knowledge and psychological support	2 (11%)	Educational in-service training and psychological support	School (1)	[59,60]

**Table 3:** Approaches to manage food allergy by type of educational setting (Total number of publications = 18).

Approaches	Setting				Total
	Childcare Centres	Preschools	Schools	Combined Settings	
Safety—Preventing allergies	0	0	2 (11%)	0	2 (11%)
Safety—Building capacities	3 (17%)	4 (22%)	5 (28%)	2 (11%)	14 (78%)
Psychological support	0	0	0	0	0
Safety and psychological support combined	0	0	2 (11%)	0	2 (11%)
Total	3 (17%)	4 (22%)	9(50%)	2 (11%)	18 (100%)

## Data Availability

Protocol registration: https://doi.org/10.17605/OSF.IO/C9E6Z. The data supporting the findings of this study are contained within the article.

## Abbreviations

The following abbreviations are used in this manuscript:

ASCIA	Australasian Society for Clinical Immunology and Allergy
CFAR	Centre for Food Allergy Research
KFA	Kids with Food Allergy
WAO	World Allergy Organization

## Appendix A. Medline (Ovid) Search Strategy (9 February 2023)

**Ovid MEDLINE(R) ALL#**	**Searches**	**Results**
1	(babies or baby or boy* or child* or girl* or infan* or juvenil* or kid* or minors or neonat* or neo-nat* or newborn* or new-born* or paediatric* or peadiatric* or pediatric* or toddler* or pre-schooler* or preschooler* or pupil* or student*).ti,ab.	3,428,898
2	exp child/or exp infant/	2,743,347
3	1 or 2	4,450,652
4	((Food* adj3 (allerg* or Hypersensitivit* or intoleran* or sensitivit*)) or Anaphyla*or Epinephrine or antigen*).ti,ab.	752,754
5	exp Food Hypersensitivity/or Anaphylaxis/or Epinephrine/or Allergens/or Antigens/	202,085
6	4 or 5	860,095
7	(((risk or safety) adj2 (assess* or prevent* or control* or evaluat* or mitigat*)) or guideline* or knowledge or protocol* or polic* or manag* or treat* or attitude* or practice*).ti,ab.	9,554,096
8	Risk Assessment/or “Risk Evaluation and Mitigation”/or Practice Guidelines as Topic/or School Health Services/or Safety Management/or Risk Management/or Disease Management/or Health Knowledge, Attitudes, Practice/or Guideline Adherence/or “Delivery of Health Care”/or Therapeutics/	766,133
9	7 or 8	9,876,455
10	(day-care or daycare or preschool* or pre-school* or child-care or childcare or nurser* or school* or early childhood or kindergarten*).ti,ab.	416,851
11	Schools, Nursery/or Child Care/or Child Day Care Centers/or Schools/	61,738
12	10 or 11	426,869
13	3 and 6 and 9 and 12	1465

## References

[1] Loh W., Tang M.L.K. (2018). The Epidemiology of Food Allergy in the Global Context. Int. J. Environ. Res. Public Health.

[2] Spolidoro G.C.I., Amera Y.T., Ali M.M., Nyassi S., Lisik D., Ioannidou A., Rovner G., Khaleva E., Venter C., van Ree R. (2023). Frequency of Food Allergy in Europe: An Updated Systematic Review and Meta-Analysis. Allergy.

[3] Osborne N.J., Koplin J.J., Martin P.E., Gurrin L.C., Lowe A.J., Matheson M.C., Ponsonby A.L., Wake M., Tang M.L., Dharmage S.C. (2011). Prevalence of Challenge-Proven Ige-Mediated Food Allergy Using Population-Based Sampling and Predetermined Challenge Criteria in Infants. J. Allergy Clin. Immunol..

[4] Peters R.L., Soriano V.X., Allen K.J., Tang M.L.K., Perrett K.P., Lowe A.J., Wijesuriya R., Parker K.M., Loke P., Dharmage S.C. (2024). The Prevalence of Ige-Mediated Food Allergy and Other Allergic Diseases in the First 10 Years: The Population-Based, Longitudinal Healthnuts Study. J. Allergy Clin. Immunol..

[5] Gawryjołek J., Krogulska A. (2021). Food-Induced Anaphylaxis in Children up to 3-Years-Old Preliminary Study. Allergol. Immunopathol..

[6] Yoon L., Kim B.R., Lee J.Y., Kim K., Kim Y.M., Kim S.H., Kim H.Y. (2017). Clinical Features of Anaphylaxis According to Age in a Single University Hospital in Korea. Asian Pac. J. Allergy Immunol..

[7] Turner P.J., Jerschow E., Umasunthar T., Lin R., Campbell D.E., Boyle R.J. (2017). Fatal Anaphylaxis: Mortality Rate and Risk Factors. J. Allergy Clin. Immunol..

[8] Stockhammer D., Katelaris C.H., Simpson M.D., Vanniasinkam T. (2022). Living with Food Allergy: What This Means for Children. J. Child Health Care.

[9] Cooke F., Ramos A., Herbert L. (2022). Food Allergy-Related Bullying among Children and Adolescents. J. Pediatr. Psychol..

[10] Fong A.T., Katelaris C.H., Wainstein B.K. (2018). Bullying in Australian Children and Adolescents with Food Allergies. Pediatr. Allergy Immunol..

[11] Polloni L., Muraro A. (2020). Anxiety and Food Allergy: A Review of the Last Two Decades. Clin. Exp. Allergy.

[12] Warren C.M., Jiang J., Gupta R.S. (2020). Epidemiology and Burden of Food Allergy. Curr. Allergy Asthma Rep..

[13] Westwell-Roper C., To S., Andjelic G., Lu C., Lin B., Soller L., Chan E.S., Stewart S.E. (2022). Food-Allergy-Specific Anxiety and Distress in Parents of Children with Food Allergy: A Systematic Review. Pediatr. Allergy Immunol..

[14] Cushman G.K., Durkin K., Noga R., Cooke F., Herbert L., Esteban C., McQuaid E.L. (2023). Psychosocial Functioning in Pediatric Food Allergies: A Scoping Review. J. Allergy Clin. Immunol..

[15] Gupta R.S., Warren C.M., Smith B.M., Blumenstock J.A., Jiang J., Davis M.M., Nadeau K.C. (2018). The Public Health Impact of Parent-Reported Childhood Food Allergies in the United States. Pediatrics.

[16] Hua X., Dalziel K., Brettig T., Dharmage S.C., Lowe A., Perrett K.P., Peters R.L., Ponsonby A.L., Tang M.L.K., Koplin J. (2022). Out-of-Hospital Health Care Costs of Childhood Food Allergy in Australia: A Population-Based Longitudinal Study. Pediatr. Allergy Immunol..

[17] Ross N., Dalke S., Filuk S., Kulbaba B., Marks D., St-Vincent J., Simons E. (2022). It Takes a Village: Perceptions of Winnipeg Parents, Students, Teachers and School Staff Regarding the Impact of Food Allergy on School-Age Students and Their Families. Allergy Asthma Clin. Immunol..

[18] Council on School Health (2016). Role of the School Nurse in Providing School Health Services. Pediatrics.

[19] Centers for Disease Control and Prevention (2013). Voluntary Guidelines for Managing Food Allergies in Schools and Early Care and Education Programs.

[20] Department for Education (2023). Allergy Guidance for Schools.

[21] Food Standards Australia New Zealand (2024). Information for Childcare Centres and Schools.

[22] ASCIA New Allergy Guidelines for Schools and Children’s Education/Care. https://www.allergy.org.au/about-ascia/info-updates/new-allergy-guidelines-to-protect-children-from-severe-allergic-reactions-in-schools-and-childcare?highlight=WyJhcyJd.

[23] Food Allergy Research and Education (2024). Food Allergy Managment in Schools (Fams).

[24] Waserman S., Cruickshank H., Hildebrand K.J., Mack D., Bantock L., Bingemann T., Chu D.K., Cuello-Garcia C., Ebisawa M., Fahmy D. (2021). Prevention and Management of Allergic Reactions to Food in Child Care Centers and Schools: Practice Guidelines. J. Allergy Clin. Immunol..

[25] Quill A., Golding M.A., Bartnikas L.M., Protudjer J.L.P. (2025). An Evaluation of Food Allergy Management Practices in a Sample of Canadian and American Schools. Nutrients.

[26] Deschildre A., Alvaro-Lozano M., Muraro A., Podesta M., de Silva D., Giovannini M., Barni S., Dribin T.E., Sandoval-Ruballos M., Anagnostou A. (2025). Towards a Common Approach for Managing Food Allergy and Serious Allergic Reactions (Anaphylaxis) at School: 2len and Efa Consensus Statement. Clin. Transl. Allergy.

[27] Santos M.J.L., Merrill K.A., Gerdts J.D., Ben-Shoshan M., Protudjer J.L.P. (2022). Food Allergy Education and Management in Schools: A Scoping Review on Current Practices and Gaps. Nutrients.

[28] Santos M.J.L., Merrill K.A., Ben-Shoshan M., Gerdts J.D., Giesbrecht D., Lavine E., Prentice S., Upton J., Protudjer J.L.P. (2023). Food Allergy Education and Management in Early Learning and Childcare Centres: A Scoping Review on Current Practices and Gaps. Children.

[29] Santos M.J.L., Riediger N., Abrams E.M., Piquemal N., Protudjer J.L.P. (2022). Elementary School Teachers’ Perceptions of Covid-19-Related Restrictions on Food Allergy Management. Nutrients.

[30] Leo H.L., Clark N.M. (2012). Addressing Food Allergy Issues within Child Care Centers. Curr. Allergy Asthma Rep..

[31] Hua T., Sambell R., Wallace R., Vale S., Devine A. (2020). Food Allergy Management in Early Childhood Education and Care Services in Australia. J. Paediatr. Child Health.

[32] Vale S., Netting M.J., Ford L.S., Tyquin B., McWilliam V., Campbell D.E. (2019). Anaphylaxis Management in Australian Schools: Review of Guidelines and Adrenaline Autoinjector Use. J. Paediatr. Child Health.

[33] Munn Z., Pollock D., Khalil H., Alexander L., McLnerney P., Godfrey C.M., Peters M., Tricco A.C. (2022). What Are Scoping Reviews? Providing a Formal Definition of Scoping Reviews as a Type of Evidence Synthesis. JBI Evid. Synth..

[34] Munn Z., Peters M.D.J., Stern C., Tufanaru C., McArthur A., Aromataris E. (2018). Systematic Review or Scoping Review? Guidance for Authors When Choosing between a Systematic or Scoping Review Approach. BMC Med. Res. Methodol..

[35] Aromataris E., Lockwood C., Porritt K., Pilla B., Jordan Z. (2024). JBI Manual for Evidence Synthesis.

[36] Pollock D., Alexander L., Peters M., Khalil H., Godfrey C., McInerney P., Synnot A., Tricco A. (2022). Moving from Consultation to Co-Creation with Knowledge Users in Scoping Reviews: Guidance from the JBI Scoping Review Methodology Group. JBI Evid. Synth..

[37] Kids with Food Allergies (KFA) About KFA. Asthma and Allergy Foundation of America (AAFA). https://kidswithfoodallergies.org/about-us/.

[38] Allergy & Anaphylaxis Australia About Us. https://allergyfacts.org.au/about-us/.

[39] National Allergy Council About Us. https://nationalallergycouncil.org.au/about-us.

[40] Australasian Society of Clinical Immunology and Allergy (ASCIA) About Ascia. https://www.allergy.org.au/about-ascia.

[41] World Allergy Organization (WAO) About Wao. https://www.worldallergy.org/wao/about.

[42] Centre for Food Allergy Research (CFAR) About. Murdoch Children’s Research Institute. https://www.cfar.org.au/about/.

[43] European Academy of Allergy and Clinical Immunology (EAACI) About Eaaci. https://eaaci.org/about-eaaci/.

[44] Asthma and Allergy Foundation of America About the Asthma and Allergy Foundation of America. https://aafa.org/about-aafa/.

[45] (2023). Endnote.

[46] Veritas Health Innovation Covidence Systematic Review Software. https://www.covidence.org/.

[47] Tricco A.C., Lillie E., Zarin W., O’Brien K.K., Colquhoun H., Levac D., Moher D., Peters M.D.J., Horsley T., Weeks L. (2018). Prisma Extension for Scoping Reviews (Prisma-Scr): Checklist and Explanation. Ann. Intern. Med..

[48] Peters M.D.J., Marnie C., Tricco A.C., Pollock D., Munn Z., Alexander L., McInerney P., Godfrey C.M., Khalil H. (2020). Updated Methodological Guidance for the Conduct of Scoping Reviews. JBI Evid. Synth..

[49] Lunny C., Pieper D., Thabet P., Kanji S. (2021). Managing Overlap of Primary Study Results across Systematic Reviews: Practical Considerations for Authors of Overviews of Reviews. BMC Med. Res. Methodol..

[50] Banerjee D.K., Kagan R.S., Turnbull E., Joseph L., St. Pierre Y., Dufresne C., Gray-Donald K., Clarke A.E. (2007). Peanut-Free Guidelines Reduce School Lunch Peanut Contents. Arch. Dis. Child..

[51] Bansal P.J., Marsh R., Patel B., Tobin M.C. (2005). Recognition, Evaluation, and Treatment of Anaphylaxis in the Child Care Setting. Ann. Allergy Asthma Immunol..

[52] Bartnikas L.M., Huffaker M.F., Sheehan W.J., Kanchongkittiphon W., Petty C.R., Leibowitz R., Hauptman M., Young M.C., Phipatanakul W. (2017). Impact of School Peanut-Free Policies on Epinephrine Administration. J. Allergy Clin. Immunol..

[53] Crow K.M. (2018). Increasing Knowledge About Food Allergy Management in the Preschool Setting.

[54] Dumeier H.K., Richter L.A., Neininger M.P., Prenzel F., Kiess W., Bertsche A., Bertsche T. (2018). Knowledge of Allergies and Performance in Epinephrine Auto-Injector Use: A Controlled Intervention in Preschool Teachers. Eur. J. Pediatr..

[55] Foster A.A., Campbell R.L., Lee S., Anderson J.L. (2015). Anaphylaxis Preparedness among Preschool Staff before and after an Educational Intervention. J. Allergy.

[56] Goertz J., Marget M., Schelling R., Persily J., Hoyt A. (2020). Epinephrine Auto-Injector Workshops Answer Allergy Questions for Early Childhood Professionals and Provide Hands-on Practice with Training Devices. J. Allergy Clin. Immunol..

[57] Lanser B.J., Covar R., Bird J.A. (2016). Food Allergy Needs Assessment, Training Curriculum, and Knowledge Assessment for Child Care. Ann. Allergy Asthma Immunol..

[58] Patel B.M., Bansal P.J., Tobin M.C. (2006). Management of Anaphylaxis in Child Care Centers: Evaluation 6 and 12 Months after an Intervention Program. Ann. Allergy Asthma Immunol..

[59] Polloni L., Lazzarotto F., Toniolo A., Ducolin G., Muraro A. (2013). What Do School Personnel Know, Think and Feel About Food Allergies?. Clin. Transl. Allergy.

[60] Polloni L., Baldi I., Lazzarotto F., Bonaguro R., Toniolo A., Gregori D., Muraro A. (2020). Multidisciplinary Education Improves School Personnel’s Self-Efficacy in Managing Food Allergy and Anaphylaxis. Pediatr. Allergy Immunol..

[61] Raptis G., Totterdell R., Gerasimidis K., Michaelis L.J., Perez-Botella M. (2021). School Allergy Training Promotes Internal Policy Review and Enhances Staff’s Preparedness in Managing Pupils with Food Allergy. Clin. Transl. Allergy.

[62] Ravarotto L., Mascarello G., Pinto A., Schiavo M.R., Bagni M., Decastelli L. (2014). Food Allergies in School: Design and Evaluation of a Teacher-Oriented Training Action. Ital. J. Pediatr..

[63] Rodriguez Ferran L., Gomez Tornero N., Cortes Alvarez N., Thorndike Piedra F. (2020). Anaphylaxis at School. Are We Prepared? Could We Improve?. Allergol. Immunopathol..

[64] Sasaki K., Sugiura S., Matsui T., Nakagawa T., Nakata J., Kando N., Ito K. (2015). A Workshop with Practical Training for Anaphylaxis Management Improves the Self-Efficacy of School Personnel. Allergol. Int..

[65] Shah S.S., Parker C.L., Davis C.M. (2013). Improvement of Teacher Food Allergy Knowledge in Socioeconomically Diverse Schools after Educational Intervention. Clin. Pediatr..

[66] Shroba J.A., McElroy S. (2019). Assessment of School Preparedness in the School-Aged Child with a Food Allergy. J. Allergy Clin. Immunol..

[67] Wahl A., Stephens H., Ruffo M., Jones A.L. (2015). The Evaluation of a Food Allergy and Epinephrine Autoinjector Training Program for Personnel Who Care for Children in Schools and Community Settings. J. Sch. Nurs..

[68] Rubeiz C.J., Ernst M.M. (2021). Psychosocial Aspects of Food Allergy: Resiliency, Challenges and Opportunities. Immunol. Allergy Clin. N. Am..

[69] Sanagavarapu P., Dadich A., Hussain W. (2023). Interventions to Promote Food Allergy Literacy in Childhood: A Systematic Scoping Review. J. Sch. Health.

[70] Pouessel G., Dumond P., Liabeuf V., Tanno L.K., Deschildre A., Beaumont P., Van der Brempt X., Beaudouin E., Labreuche J., Renaudin J.-M. (2019). Gaps in the Management of Food-Induced Anaphylaxis Reactions at School. Pediatr. Allergy Immunol..

[71] Sdona E., Turesson A., Zelander C.F., Lövquist A., Lauber A., Georgelis A., Bergström A., Jonsson M. (2023). Management of Children with Allergies in Preschool and School-Potential for Improvements. Pediatr. Allergy Immunol..

[72] Nielsen W.W., Lindsey K. (2010). When There Is No School Nurse--Are Teachers Prepared for Students with Peanut Allergies?. Sch. Nurse News.

[73] LeBovidge J.S., Herbert L.J., Ramos A., Rotter N., Sicherer S.H., Young M.C., Pistiner M., Phipatanakul W., Bartnikas L.M., Bingemann T.A. (2022). The Development of Age-Based Food Allergy Educational Handouts for Caregivers and Patients: A Work Group Report of the AAAAI Adverse Reactions to Foods Committee. J. Allergy Clin. Immunol. Pract..

[74] Tsuang A., Wang J. (2016). Childcare and School Management Issues in Food Allergy. Curr. Allergy Asthma Rep..

